# An mHealth-based school health education system designed to scale up salt reduction in China (EduSaltS): A development and preliminary implementation study

**DOI:** 10.3389/fnut.2023.1161282

**Published:** 2023-04-17

**Authors:** Puhong Zhang, Jingwen Sun, Yinghua Li, Yuan Li, Yuewen Sun, Rong Luo, Xueqiong Nie, Li Li, Yu Liu, Feng J He

**Affiliations:** ^1^The George Institute for Global Health, Beijing, China; ^2^Chinese Center for Health Education, Beijing, China; ^3^School of Computing Science and Engineering, Beihang University, Beijing, China; ^4^Barts and The London School of Medicine and Dentistry, Wolfson Institute of Population Health, Queen Mary University of London, London, United Kingdom

**Keywords:** school-based health education, salt reduction, behavior change, intervention platform development, m-health, prevention

## Abstract

**Background:**

High-salt diet is an important risk factor for several non-communicable diseases. School-based health education has been found effective in reducing salt intake among children and their families in China. However, no such interventions have been scaled up in the real world. For this purpose, a study was launched to support the development and scale-up of an mHealth-based system (EduSaltS) that integrated routine health education and salt reduction and was delivered through primary schools. This study aims to elaborate the framework, development process, features, and preliminary scaling-up of the EduSaltS system.

**Methods:**

The EduSaltS system evolved from previously successfully tested interventions to reduce family salt intake by empowering schoolchildren through school health education. EduSaltS was designed by following the WHO’s conceptual framework for developing a scaling-up strategy which accounted for the nature of the innovation, the capacity of the implementing organizations, the characteristics of the environment, the resources available, and type of scaling up. The system was then developed step by step from determination of online platform architecture, definition of component interventions and activities, development of specific educational materials and tools, to the development of the online/offline hybridized system. The system was tested and refined by a pilot in two schools and a preliminary scale-up in two cities in China.

**Results:**

EduSaltS was developed as an innovative health education system, including an online WeChat-based education platform, a set of offline activities, and an actual administrative website showing the progress and setting the system. The WeChat platform could be installed on users’ smartphones to automatically deliver 20 sessions of five-minute well-structured cartoon video classes, followed by other online interactive activities. It also helps support project implementation and real-time performance evaluation. As a first-stage roll-out, a one-year course has been successfully implemented among 54,538 children and their families from 209 schools in two cities, and the average course completion rate was 89.1%.

**Conclusion:**

As an innovative mHealth-based health education system, EduSaltS was developed based on successfully tested interventions and an appropriate framework for scaling up. The early-stage roll-out has shown its preliminary scalability, and further evaluation is ongoing.

## 1. Introduction

There is strong evidence that excessive dietary salt consumption is the major cause of raised blood pressure (BP) and an important risk factor for several non-communicable diseases (NCDs) (1–3). Reducing population salt consumption is identified as one of the best-buy preventive strategies by the World Health Organization (WHO) (3). China is one of the countries that have the highest salt intake in the world, with an average of 11 g/day (4), and a high-salt diet led to over 1.5 million deaths each year in China (5). Although various multifaceted salt reduction studies have been undertaken for many years in China (6–12), the progress on reducing population salt intake in the past 40 years was not satisfactory (13).

Different from western countries where most salt consumption comes from prepackaged food, 75–80% of the salt in the Chinese diet comes from salt or other salty condiments added during cooking (1). Instead of setting incrementally lower salt targets for processed foods like western countries (14), China has to focus on reducing discretional salt use during cooking, which is a huge challenge because individual cooking behaviors are difficult to change (15).

The disciplined and cooperative structure of schools make it more likely for students’ parents to comply with the requirements of school for knowledge learning and behaver change. This health education through schools has found useful in helping parents stop smoking (16), improve cardiovascular health (17) and increase physical activity and lose weight (18). In China, children seem to have bigger influences on their families due to the implementation of ‘one-child policy’ for many years from 1982 to 2016. Not surprisingly, the strategy of “small hands leading big hands” was also found effective in reducing salt intake in both children and adults and lowering BP in adults by a cluster randomized controlled trial (cRCT) called School-EduSaltS in 2015 (11, 19, 20).

Several attempts such as primary care delivered intervention and specially designed salt spoon were also introduced to reduce salt use at home, but their effectiveness has not been supported by robust evidence (21, 22).

Although found effective in salt reduction by RCT, the “small hands leading big hands” strategy delivered by School-EduSaltS is not easy to be rolled out. It was delivered through a specially designed offline course and a set of activities to engage schools, children, and parents, which needs continuous training and organization for the implementers (who usually were the school teachers). To reduce school teachers’ workload, an mobile Health (mHealth) supported platform (AppSalt) was developed to provide schoolchildren and their parents with a standardized online health education course and parallel homework activities. Evaluated by another cRCT called AIS (12, 23), the AppSalt platform was also found to be effective in lowering salt intake among adult participants (4).

However, the mHealth-based AppSalt was still faced with some major scaling issues. Firstly, the electronic course was in the form of recorded videos of classroom presentation which were not attractive to the students. Each lesson was at least 10 min long, and parents worried that it might damage children’s eyesight. Second, although the app was developed based on iOS and Android, it was still difficult to adapt to various mobile phone brands, versions, and operating systems. Thirdly, there were some children who lived with their grandparents and had limited access to smartphones. Finally, but most importantly, there were less administrative modules in AppSalt such as user management and performance evaluation to support routine project management, which would be a big barrier to the delivery of the innovative course in the real-world where the health education course is usually overlooked by primary schools (24).

In summary, the previous interventions are not attractive, compatible, flexible, and supportive enough to be scaled up at different settings. To further roll out this promising and novel approach to a larger population, a development and preliminary implementation study named EduSaltS programme was launched to further update the mHealth platform, and roll it out in piloting areas of China and then nationwide (25). Until now, “small hands leading big hands” is one of the few strategies that are effective in salt reduction mainly targeting discretional salt use in China (4, 19, 26–28). In theory, if mandatorily implemented by the Chinese government, this intervention could cover the whole country in a relatively short time. This would be of great significance for achieving China’s goal of reducing salt intake by 20% by 2030 (29).

This study aims to elaborate the framework, development process, features, and preliminary implementation of the EduSaltS system, with the purpose of enhancing the understanding of this project and providing reference for researchers/developers when designing mHealth-based interventions for scaling up.

## 2. Methods

The EduSaltS programme is a one-arm implementation study to scale up the successfully tested interventions (School-EduSaltS and AppSalt) developed by our previous cRCTs: School-EduSalt study (11, 19) and ASC-AIS study (4, 12). The EduSaltS programme follows a four-phase model of implementation spanning preparation, pilot, scale-up and sustainment (30). At the preparation and pilot phase, an innovative mHealth-based primary school health education system containing salt reduction content (EduSaltS) was developed and piloted. At the scale-up phase, at least 100 primary schools would be enrolled from each of the three prefecture-level cities in South (Ganzhou, Jiangxi Province), Central (Zhenjiang, Jiangsu Province) and North China (Qinhuangdao, Hebei Province) to evaluate the real-world implementation of EduSaltS at different settings including urban and rural areas. The overall design of EduSaltS programme and the two underpinned cRCTs are summarized in Supplementary Table 1.

**Figure 1:**
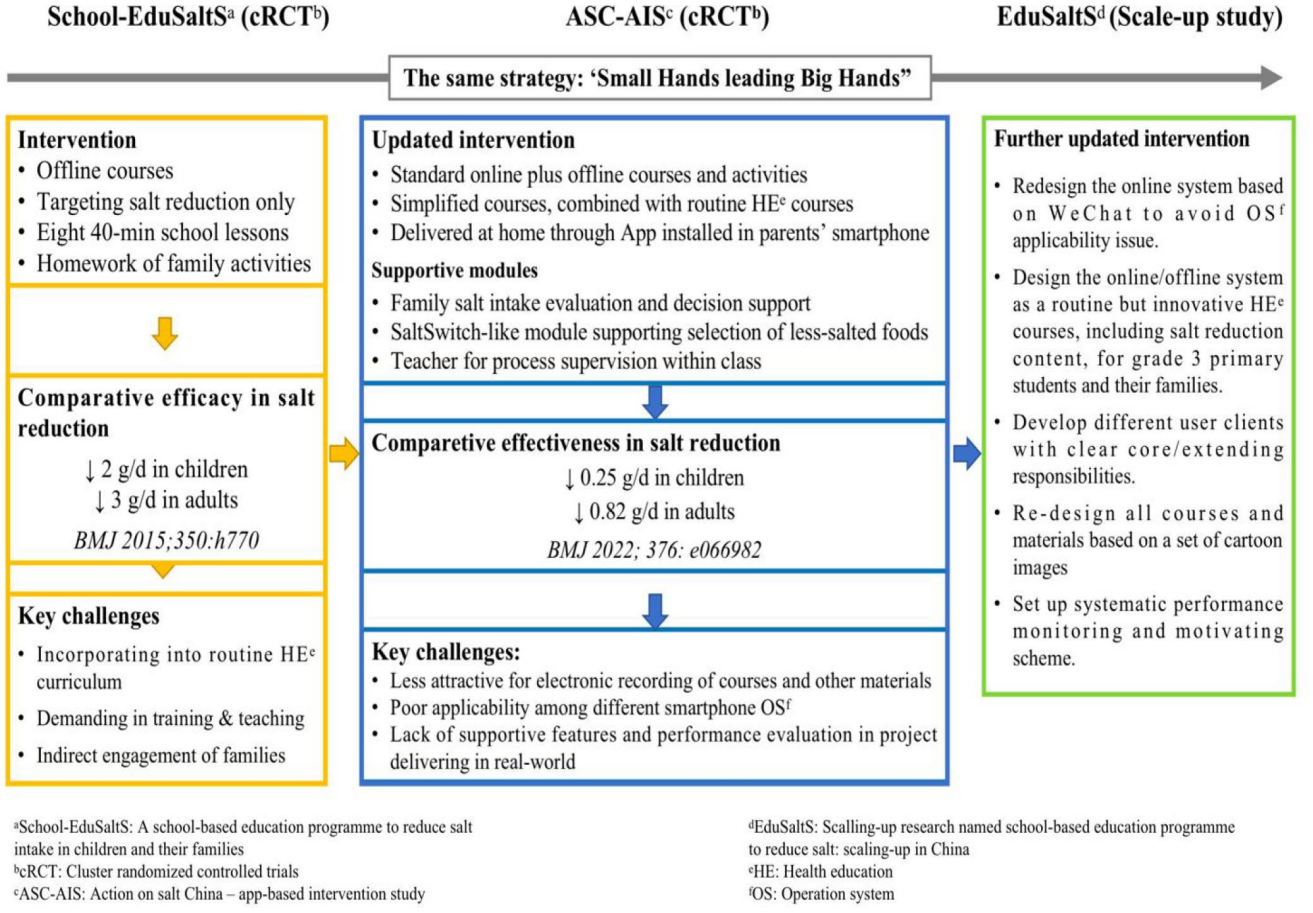
The evolution of interventions from School-EduSalt, AppSalt to EduSaltS.

Evolved from the offline intervention of School-EduSalt and mHealth-based AppSalt, the EduSaltS system was developed and updated following the five steps: (1) determining the overall framework, (2) setting the IT architecture for the online platform, (3) defining the component interventions and activities, (4) developing education materials, online system and offline activities, and (5) pilot use and system refinement. The evolution of the interventions as well as the major difference between them are summarized in Figure 1.

### 2.1. Overall framework

We outlined a guidance framework to instruct the development of EduSaltS system based on the WHO’s conceptual framework for developing a scaling-up strategy which accounts for the core elements including the nature of the innovation, the capacity of the implementing organizations, the characteristics of the larger environment within which scaling up takes place and the resources available to support the process, as well as some strategic choice areas relevant to the type of scaling up (31).

The framework is the foundation when developing the scaling up system. The detailed attributes to each of the core elements and strategic choice areas considered during the development of EduSaltS are summarized in Supplementary Table 2. In summary, EduSaltS is an mHealth-based primary school health education system which can (semi-)automatically deliver routine health education course by engaging the schools, students and their families in the course and parallel online/offline activities with the purpose of improving their health knowledge and behaviors including salt reduction in China.

Two other key aspects are worth mentioning. As adopted in AIS project, the target population is still grade-3 students (age 8–9 years) and their families because the grade 3 students are mature enough to understand the knowledge of salt reduction and deliver relevant information to their families and have less study load compared with other age groups. The online platform would be developed based on WeChat, the most popular social network platform in China which has already been adapted to smartphones of almost all brands, versions, and operating systems. It can be easily used by scanning a QR code or searching the Chinese name of the application on WeChat, without considering compatibility issues encountered by traditional smartphone applications when being (re-)installed, used, and upgraded.

### 2.2. Architecture of the online platform

EduSaltS system contains online and offline activities. According to the guidance framework, the architecture of the online platform should meet some key requirements.

1. It can support both online and offline health education courses and activities. This means that the online platform will not only provide online lessons and activities, but also have modules or embedded features which can enable users to easily access abundant materials and designs used for offline activities and to collect information about offline activities for online assessment and performance evaluation (See Supplementary Figure 1, for example, screenshots of online and offline connection).
2. It can be registered and launched by schools at any time (usually at the beginning of each school year) in different regions. This requires that the user architecture should be developed following China’s administrative divisions, and the local governments can be authorized to invite their subordinated schools to join the course and to set the starting date of the course through a website (See Supplementary Figure 2, for example, screenshots of user and school management).
3. It can play different roles for different users, such as e-learning for students and their families, and process supervision for teachers in charge of classes, school heads and government officers. This means that at least two application clients for student/family and the supervisors are needed and should be developed as per their roles and responsibilities.
4. Supportive modules for mandatory/optional use should be available for different users at different settings in order to strengthen knowledge learning and motivate behavior change. Modules like knowledge competition, salt intake monitor and performance ranking, and features supporting offline learning and activities should be designed to improve users’ Knowledge, attitude and practice (KAP) and self-efficacy. However, all the features and resources should be clearly noted as mandatory or optional to improve the system’s flexibility and acceptability (See Supplementary Figure 3 for example screenshots of optional or mandatory tasks for a teacher).
5. It can automatically record and report the progress and performance of different users in real time to make sure that the innovative course can be delivered in time with high quality. This requires all key activities, online or offline, can be recorded and properly scored. Key progress information and evidence of offline activities could be uploaded to the system using structured form by responsible users (See Supplementary Figure 1, for example, screenshots of online and offline connection and Supplementary Figure 4 for the general algorithms scoring activities).

### 2.3. Determination of component interventions and activities

Following the framework and architecture of EduSaltS, based on the experience from previous studies, and referring to the established behavior change theories including the KAP theory (32–34), the health belief model (HBM) (23, 35, 36), and social networks (37), we defined the specific intervention components and activities for EduSaltS to build healthy and supportive campus environment, and enhance individual knowledge and belief, skills, practice, engagement and communication for participants including children, families, teachers and organizers. The intervention components and activities of EduSaltS are summarized in Table 1.

**Table 1:** Outline interventions and activities of EduSaltS.

Intervention/activities		Description	Users/places
Online intervention platform			
	Health education course	20 cartoon style health education lessons with quizzes and practices; five minutes each, 12 for routine health education^b^, and 8 for salt reduction.	Student and family
	Other supportive features	Library of various education materials designed for different populations and settings; knowledge competition; family salt intake monitor (38); FoodSwitch module for healthy food selection (39); canteen food evaluation; Q&A and communication; scoring and promotion.	Student and family
Offline activities^a^			
	School environment building	Healthy environment building, with clear requirements, data and material support, evidence collection, and performance evaluation	School, class and canteen
	Other interactive activities in schools	Encouraging schools/classes to organize various interactive activities reinforcing the health education, such as health knowledge competition, class meeting, parent meeting, etc.	School, class, and family
		Most were optional with designated purpose and design.	
	Administrating activities	Administrating activities including political supports, online monitoring *via* informatization tools and offline monitoring *via* on-site supervision	Government administrators
Management and evaluation system			
	App-based management	User administration, online/offline monitoring and feedback, technical support, evidence collection for offline activities, and education materials downloading.	School and government administrators
	Web-based management	School recruitment, role setting, system setting, announcement of temporal notice and activities, evidence confirmation, performance summary and specific statistics.	Government administrators

a The offline activities are also supported by online features such as sample designs, supportive materials, process regulation, evidence collection and scoring for performance evaluation.

b The routine health education for grade 3 students includes eyesight protection, healthy lifestyle, healthy sleeping, and COVID-19 prevention and control, etc.

### 2.4. Development of education materials, online system, and offline activities

Educational materials were developed from previous School-EduSalt and AppSalt based on KAP, HBM and social network theories, but redesigned based on a set of uniformed cartoon characters to improve their attractiveness. This work was co-led by The George Institute and the Chinese Center for Health Education (CCHE). All the materials, in form of hardcopy, audio and video, designed for children and adults in different settings, were developed under the procedures required by the Guide to the Generation and Dissemination of Popular Health Science Information (40), and incorporated into the Resource Bank for Health Promotion and Health Education of CCHE (41) (See Supplementary Table 3 for the key components of the health education materials).

The online platform was developed by the IT team of Beihang University under the support of the research team following the pre-determined architecture, interventions, and activities. It contained WeChat applications and a website used for lessons delivering, process and progress monitoring, performance evaluation, high-level administration, and system setting. Data, algorithms and materials used in developing different functions or features were provided by the research team.

The offline activities included (1) administrative activities such as policy support, announcement release and online/offline supervision, (2) school activities such as school environment building, other school-level health promotion activities, and salt reduction *via* school canteen, and (3) class activities such as offline health education course and other health education activities. Recommended design, frequency and reporting formats for these activities have been illustrated in the management client and in the operation manuals designed for administrators, teachers, and families.

### 2.5. Pilot use and system refinement

A pilot study was conducted in Zhenjiang due to its geographical location (i.e., in central China which is more representative compared with that in other part of China) in September 2020 to test the feasibility and adaptability of the scaling-up packages. A total number of 172 participants in two schools (1 urban school and 1 rural school) attended this one-month pilot study. Participants were provided with four cartoon health education lessons and other parallel online and offline activities through the EduSaltS system after one training seminar organized by the local health authorities in each school. At the end of the pilot study, individual and focus-group interviews were conducted among 1 local education bureau officer, 1 local health bureau officer, 4 specialists on health education from local provincial and city level CDC (also the local leaders of the project), 2 schoolmasters, and 2 teachers in charge of classes. Four focus-group interviews were also conducted among 8 schoolchildren and 8 parents separately with the support of the two piloting schools (See Supplementary text 1 for the interview outlines). The challenges and solutions based on the pilot study are summarized in Supplementary text 2. The three key aspects were: (1) to improve family engagement by interactive activities such as parents’ meeting, and providing hardcopy and audio educational materials for the families who were unable to use smartphone application; (2) to empower children by parallel health education course at school, homework of participating family activities such as cooking to understand salt reduction, and performance ranking among classmates; and (3) to reduce the burdens and increase the feasibility of EduSaltS by outlining the key responsibilities for each role of student/family, teacher and administrator, and clearly marking activities as mandatory or optional to reduce the burden and increase the flexibility of the programme.

The development process was reported following the Guidance for Reporting Intervention Development Studies in Health Research (GUIDED) checklist (42).

### 2.6. Ethical considerations

The pilot and main study of EduSaltS programme was approved by Queen Mary Ethics of Research Committee (QMERC2020.033) and the Medical Ethical Review Committee of Chinese Center for Health education (2020003). The main study was registered in Chinese Clinical Trial Register (ChiCTR2000039767). Written informed consent from all participants was obtained before their participation in the programme. For children, written consent was obtained from children themselves as well as their guardians. All participants will be free to withdraw from the programme at any time with no explanation required. De-identified data can only be used for statistical analysis and report.

## 3. Results

**Figure 2:**
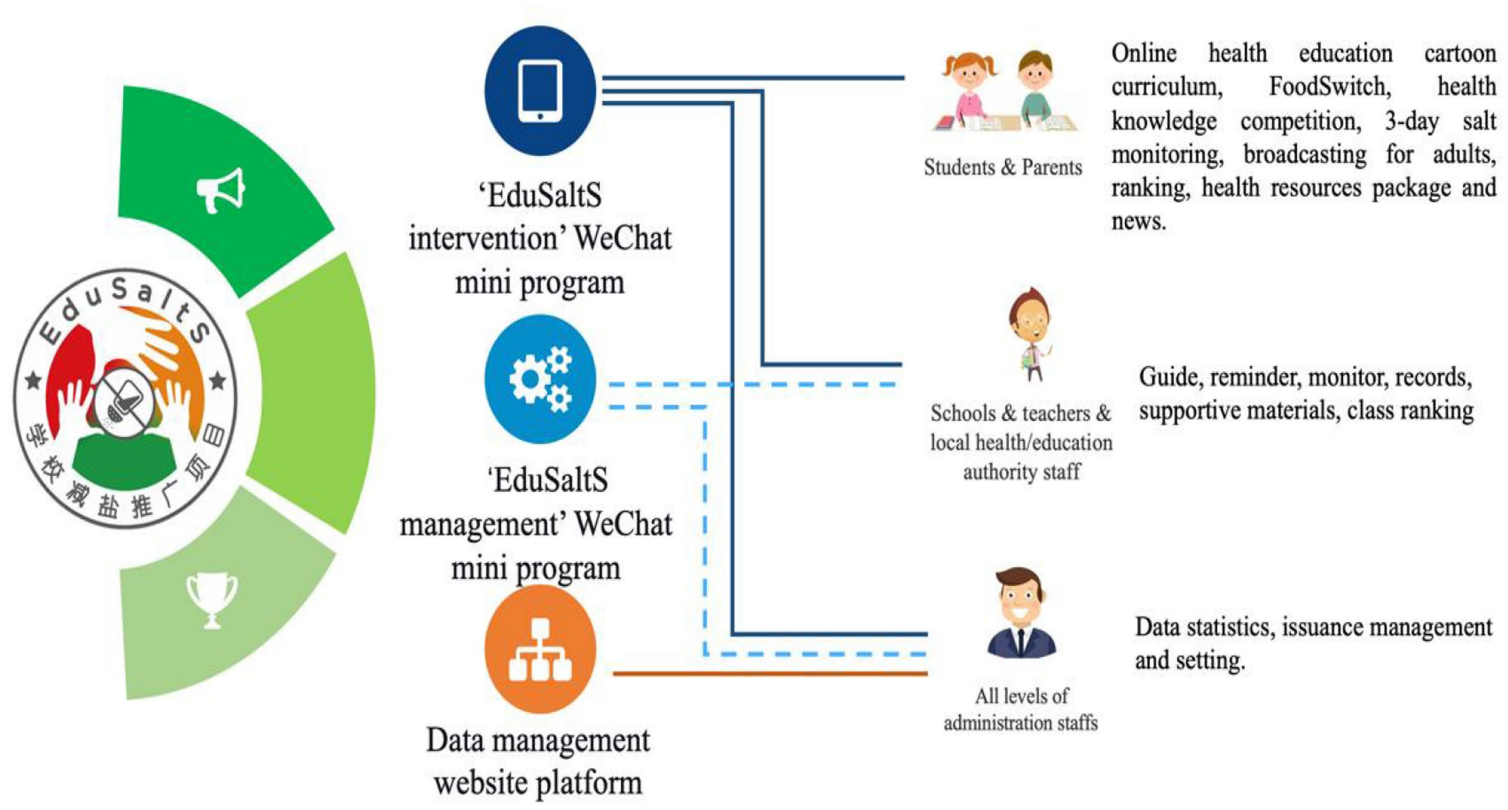
Three electronic components of the ‘EduSaltS’ system.

The final output is an innovative health education system covering a standard health education course and supportive features and activities facilitating knowledge learning and behavior change, especially for salt reduction. The system is composed of a WeChat platform supporting online activities, a set of app-based offline activities, and a website supporting system setting and performance evaluation (Figure 2). The WeChat platform contains two WeChat apps named ‘Health Cloud Classroom’ (健康云课堂)—the client for children/parents and ‘EduSaltS Manager’ (健盐管理端)—the client for teachers and supervisors which can be easily installed after scanning their QR codes or searching their Chinese names in WeChat.

### 3.1. ‘Health cloud classroom’

**Figure 3:**
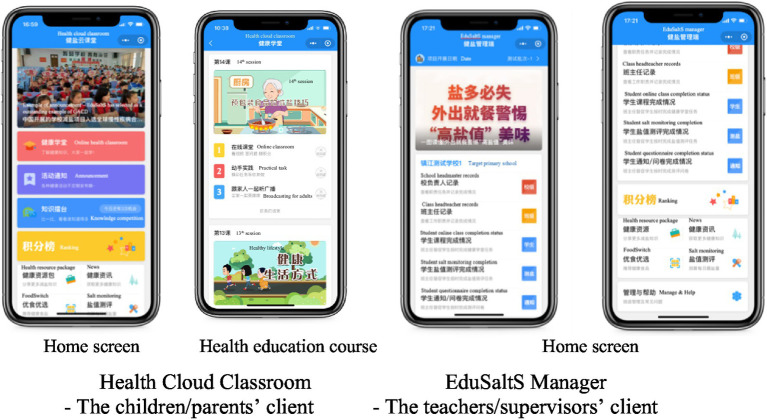
The main features of ‘Health Cloud Classroom’ and ‘EduSaltS Manager’.

‘Health Cloud Classroom’ is a WeChat app designed for students and their families. Its main function is to deliver a cartoon health education course followed by some supportive features (Figure 3).

The health education course has 20 cartoon lessons covering salt reduction and other health topics (Supplementary Table 3). Each lesson starts with a 5-min cartoon class and ends with a summary, followed by 10 quizzes. The whole course lasts for 1 year (two school terms) with a lesson automatically released per week. A KAP examination is conducted before and after the course to evaluate the performance of each student.

The supportive features are some interesting activities and easy hands-on practices developed to enhance the KAP, belief and social network. ‘Announcement’ (活动通知) releases news about temporarily organized activities such as an art competition, a KAP questionnaire, and saltiness evaluation survey on dishes provided by school canteens. ‘FoodSwitch’ (优食优选) is a module to help users to make better pre-packaged food choices, especially those with less salt. ‘Knowledge Competitions’ (知识擂台) is a game-like knowledge competition among classmates to cultivate interest in learning salt-related knowledge and other health knowledge. There are 6 questions per round. Each student can participate in maximum of three rounds per day. ‘Salt Monitoring’ (盐值测评) is a tool to estimate salt intake for all family members based on household consumption of cooking salt, salted condiments and prepackaged foods within 3 days. It can estimate the amount and source of salt intake and suggest corresponding salt reduction actions. ‘Ranking’ (积分榜) automatically collects the pre-assigned scores for completed activities and ranks the total scores for individuals among classmates in a class and school. Participants who reach a score threshold will be awarded with an electronic animal badge as an encouragement. Users can also view the details of their points and badges obtained. ‘Health Education Package’ (健康资源包) is a collection of all education materials in electronic version for free use (Supplementary Table 4). ‘News’(健康资讯) is a collection of news and press release which shows significant progress, good experience and new requirements of EduSaltS. The items in the ‘News’ are scrolled on the home page of ‘Health cloud classroom’. ‘Broadcasting’ (跟家人一起听广播) is a module newly added after the pilot study to help (grand-)parents and children gaining key message of the course without watching the small screen of smartphone. There are eight audio broadcastings matched with the salt reduction cartoon lessons.

### 3.2. ‘EduSaltS manager’

‘EduSaltS Manager’ is designed for teachers, school principals and government administrators to support subordinated progress supervision, offline activity implementation, evidence collection, and performance evaluation. The modules shown in ‘EduSaltS manager’ differ for different users. Overall, the functions include responsibility statement and mandatory/optional task list, formatted data collection showing the implementation and quality of online/offline activities, performance evaluation, and feedback for queries and requirements. Some tools included in ‘Health Cloud Classroom’ such as ‘Health Education Package’, ‘News’, ‘FoodSwitch’ and ‘Salt Monitoring’ are also embedded in the ‘EduSaltS Manager’ (Figure 3).

### 3.3. Offline activities

Offline activities are designed to facilitate the online course and activities, but the offline activities are also supported by the online platform through recommended design and downloadable materials (Supplementary Table 4).

Offline health education course: Considering the potential difficulties faced by the parents in using smartphone, we provided each school with a set of teaching materials which can be used as a substitution or supplement to the online course. These include posters, slides and cartoon videos, and so on. Each school can design its own teaching plan but it must cover all the health topics and coincides with the progress of the online course.

Health education activities: Several offline school/class activities are designed for school/class to reinforce knowledge and facilitate behavior change, including parent meeting, salt reduction knowledge competition, salt reduction art creativity activity, and salt reduction composition competition. Students who perform outstanding in these activities can be awarded with prizes, certificates or prizes to encourage their participation.

Environment building: Provided with various education materials such as stickers, posters, broadcast audios and short films, schools and classes are required to present the materials at different settings at least once in a school term to enhance the health environment.

Salt reduction *via* school canteens: We have specially designed an application for salt reduction training for school canteen chefs. In addition, the system can also release the salinity evaluation of canteen dishes through the “Announcement” module of the “Health Cloud Classroom” occasionally to guide chefs and students to pay attention to reducing salt. The evaluation results will be fed back to the school chefs to improve their salt reduction awareness and compliance. We recommend that all school chefs participate in this activity. The headmasters can monitor the process through “EduSaltS Manager.”

### 3.4. EduSaltS management website

**Figure 4:**
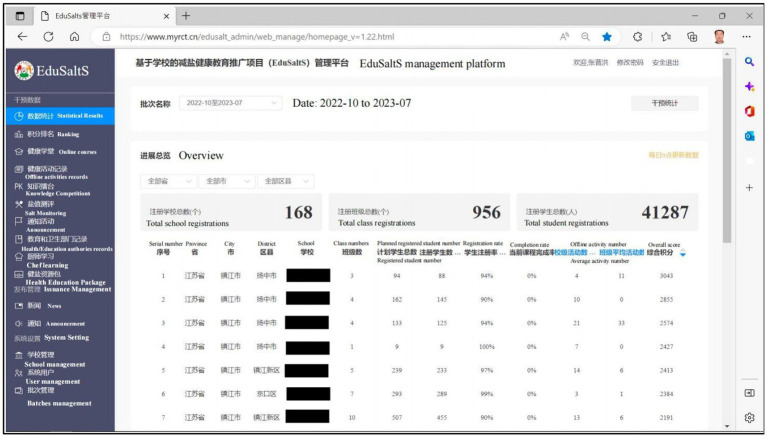
The web based of EduSaltS management platform for statistical reports and system setting.

A website named EduSaltS management platform (43) (Figure 4) is designed for all-level administration officers to support the programme management, including the following three functions:

‘Statistical Results’ (统计数据): All-sided data collected from the WeChat platform is summarized by this module on the home page, including statistics for online classes, offline activities, ranking, knowledge competitions, salt monitoring, and documents uploaded by Health/Education authorities. Quality assessment is specially designed for the national administration officers to evaluate the records of offline activities records submitted by classes, schools, and local administration officers. Health resource package is also available for free download and use in online and offline activities.
‘Issuance Management’ (发布管理): Used by the national administration officers, the main function is to edit, manage and issue the news and announcement of the programme.
‘System Setting’(系统设置): In this module, national or other authorized administrators can set up accounts for subordinated administrative persons in charge of schools and classes, in order to generate code for student registration. It can also help launch the innovative health education course for the subordinated schools by simply setting the starting date of the course.

### 3.5. Comprehensive performance evaluation

Comprehensive performance evaluation system is a practical tool to award outstanding students, teachers, and schools. Children can obtain credits from the online activities they complete and offline activities organized by teachers or schools. The teachers’ score is the average of the children’s scores in the class. Considering that organizing offline activities may increase the burdens on teachers, we give more credits for offline activities and automatically be counted after the completion. Users could clearly see the results displayed on all platforms (Supplementary Figure 4).

### 3.6. Current use of EduSaltS system

EduSaltS was planned to roll out in two phases: phase 1—a smaller scale promotion in 60 schools (20 for each of the 3 participating cities) to further resolve potential issues found during scaling-up, and phase 2—a larger scale promotion among the remaining 240 schools (80 for each city). Until Dec 15, 2022, both Ganzhou and Zhenjiang had completed phase 1 and proceeded with phase 2 scale-up. In total, 54,538 students and their families from 1,262 classes of 209 schools had been covered by the two-phase intervention. The registration rate of students was 97.8% (54,538/55,762), and the completion rates of the 20 cloud lessons were 89.1% on average, from 82.3 to 94.6%. Offline activities organized by schools and classes was full of variety, with median of 5 (interquartile range: 1–10) school-level activities per school, and median of 3 (1–6) class-level activities per class among the 306 classes of 41 schools who had completed the phase 1 course. The offline activities included posters, health education course or activities, salt reduction training for chefs, and knowledge competition and rewards. About 11,570 students completed the baseline and end KAP evaluation, with the KAP score increased from 74 to 83 out of 100. However, the scaling-up had not been launched in Qinghuangdao due to the epidemic of COVID-19.

During the phase 1 scale-up, we further found that many participants did not understand the programme very well, so we developed a 3-min video introducing the programme before the first class with the purpose of raising their awareness and adherence to this innovative intervention. To facilitate communication and supervision, a feature was also developed to automatically send a short message through WeChat to inform the head teachers and managers at all levels of the completion rate of the lessons of their classes or schools.

## 4. Discussion

### 4.1. Principal findings

EduSaltS was developed as an innovative mHealth-based health education system delivered through primary schools to improve the health knowledge and behaviors of schoolchildren and their families including salt reduction. It was evolved from previously tested interventions based on behavior change theories, upgraded following the WHO’s theoretical framework for developing a scaling-up strategy (31) and continuously refined based on the findings from pilot use and scaling-up.

### 4.2. Advantages of EduSaltS

Compared to the underpinned interventions, EduSaltS has many advantages to make it more compatible, attractive, efficient, flexible and supportive, which in turn would benefit its scale-up (1). EduSaltS online platform is developed based on WeChat, which is the most popular social platform in China. The widespread use of WeChat speared the researchers from the compatibility issues of the mHealth tools for scaling up (2). All the health education materials, whether in pictures, audio, or video, were redesigned based on user age group, for example, using uniformed cartoon figures for children to improve the system’s attractiveness and users’ adherence. Many other modules or features like knowledge competition, ranking, animal badges, and participation in home cooking were also used for the same purposes (3). The EduSaltS platform can automatically deliver a standardized and high quality health education course in the absence of qualified health education teachers and textbook, which can reduce the burden of a school in delivering such a course required by the government and it can help establish the healthy image of a school (44). From the perspective of parents, the resistance is possibly low because the course would benefit the health of their children and their own (4). Except for the core lessons and matching activities, there are abundant materials and well-structured activity designs for optional use, online or offline (5). There are many supportive features for practical use. For example, the user architecture was designed according to China’s administrative divisions which can ensure that the schools in a district or a city can participate in the system at any time. The progress/performance evaluation can be implemented automatically in real-time which can largely reduce the burden of routine management.

Based on the strategy of “small hands leading big hands,” the health education implemented as a school course in one or two school terms could empower the schoolchildren with the health knowledge and skills for the dissemination to all children’s families. This will help achieve a rapid and wide coverage of the interventions in a short time. In addition, the mHealth-based system is particularly convenient to be implemented during the epidemic of COVID-19. That is why even during the most severe period of epidemic control when most offline activities were forbidden, the EduSaltS still covered almost all the target schools, students, and families in the piloting cities, with very high registration rate and course completion rate.

### 4.3. Comparison with prior work

Compared with our previous salt reduction interventions through schoolchildren carried out in China (11, 12), we paid more attention to sustainability and applicability during the development and updates of EduSaltS, such as combining salt reduction with routine health education course, developing apps based on WeChat, providing various functions and core/extending responsibilities for different users, and real-time performance evaluation. These are important factors when implementing a programme in the real world (45).

A Digital Education to Limit Salt in the Home (DELISH) program was conducted *via* website for 5 weeks by researchers from Australia (46, 47). This program generated similar findings with EduSaltS and supported that children could actively participate in weekly Web-based sessions while parents would receive concurrent educational materials through online newsletters sent *via* email and short messages with a central study website providing all resources. DELISH was very comprehensive but further efforts are needed to align with the school curriculum and the school’s teaching program (48).

The Shandong Ministry of Health Action on Salt Reduction and Hypertension (SMASH) was a collaboration action co-led by the Chinese Ministry of Health and the Shandong provincial government (7). SMASH was a comprehensive initiative covering the whole province in health education and various environment-specific interventions including school salt reduction. Unlike EduSaltS that combines online features and offline activities, SMASH only provided traditional health education classes on salt reduction for children in school, which has similar challenges found in School-EduSalt study (4).

In China, a recent cRCT proved the effectiveness of a novel multifaceted mHealth-based intervention for obesity prevention (49). In this study, a smartphone app was a tool to assist the intervention by strengthening family involvement and monitoring children’s key health indicators, but as our previous School EduSalt project, this study also delivered the key health education course by trained teachers in school (49), which is an additional task needs further consideration (24).

Several other studies using mobile phone apps to promote healthy salt reducation are under way or have just completed their pilot work, however, none of the interventions were mediated by school students (22, 50, 51).

### 4.4. Further analysis and evaluation

Several evaluations will be conducted to inform and facilitate future national scale-up and international adaptation of EduSaltS. (1) Seven hundred and eighty children and their families have been randomly selected to assess the before-after change on KAP of salt-reduction and 24 h urinary sodium excretion. RCT design was not adopted because the efficacy/effectiveness of such intervention has been confirmed by two previous RCTs (4, 19). (2) About 80,000 participating students will receive a standardized test before and after the course to examine their overall performance, in addition to KAP changes. (3) Process evaluation will be conducted based on quantitative data routinely collected by EduSaltS system and qualitative findings by individual and focus-group interviews to assess the uptake rates of the scale-up package as well as the facilitators and barriers to implementation.

### 4.5. Public health implications

The successful scale-up of EduSaltS in China may have major public health implications to many Asia, Africa and South America countries where dietary salt mainly comes from salt added during cooking or at table (52–56), and food reformulation by setting sodium targets for prepackaged foods has limited impact on salt reduction in this scenario. Given the high coverage of primary education worldwide, intervention like EduSaltS can cover both school children and their families in a very effective and efficient manner. Conceivably, the scale-up of such intervention would play a huge role in salt reduction worldwide.

The features developed in EduSaltS, the experience learnt from its real-world scale-up, and the barriers and facilitators to be found later could be a good reference for other countries if similar “children to parents” strategy would be adopted to reduce salt intake or deal with other public health issues. As part of Global Alliance for Chronic Diseases (GACD) implementation science programme, EduSaltS could inform the efforts of NCD prevention and control in many countries, particularly LMICs. In fact, EduSaltS has been selected one of ten case studies by independent evaluation to highlight the impact of GACD projects (57).

### 4.6. Limitations

Due to the limited time and funds, the materials and system functions remain further optimization. For example, the function of 3-day salt monitoring was not smooth enough, which was prone to data omission, with reminding function and quality assessment to be added. As implementation research and to make it secure enough, the admission of participating cities and schools was under the unified control of the national team. In the future, such authority should be transferred to local government, so that the system can provide the service for any schools at any time. This function has been implemented technically in EduSaltS, but it may require further improvement in security level and computing power of the server.

## 5. Conclusion

EduSaltS is an expanded innovative mHealth-based primary school health education system, which was designed based on successfully tested intervention, appropriate framework, and integrated existing resources for various users at different settings. It combines multiple functions together, including fun online learning and interactive offline activities, all-level management, and real-time evaluation. The current progress has shown its preliminary scalability by its broad coverage and high completion rate, and the development process of such a system can be very helpful for other similar studies and work.

## Data availability statement

The raw data supporting the conclusions of this article will be made available by the authors, without undue reservation.

## Ethics statement

The studies involving human participants were reviewed and approved by Queen Mary Ethics of Research Committee (QMERC2020.033) and the Medical Ethical Review Committee of Chinese Center for Health education (2020003). Written informed consent to participate in this study was provided by the participants’ legal guardian/next of kin.

## Author contributions

PZ and FH conceived the project and contributed equally to the funding application. PZ, YiL, and YuL led the design and development of the system and materials, with support from all the authors. JS and PZ contributed equally to this paper and share first authorship. JS oversaw the routine development of the intervention. YLi as the IT developer, led the development and maintenance of the online platform. All co-authors reviewed the manuscript, gave comments, and approved its submission.

## Funding

This work was supported by the Medical Research Council [MR/T024399/1], under the Global Alliance for Chronic Disease (GACD) Scale-up Research Programme.

## Conflict of interest

FH is an unpaid member of Action on Salt and World Action on Salt, Sugar and Health (WASSH).

The remaining authors declare that the research was conducted in the absence of any commercial or financial relationships that could be construed as a potential conflict of interest.

## Publisher’s note

All claims expressed in this article are solely those of the authors and do not necessarily represent those of their affiliated organizations, or those of the publisher, the editors and the reviewers. Any product that may be evaluated in this article, or claim that may be made by its manufacturer, is not guaranteed or endorsed by the publisher.

## Abbreviations

AIS: app-based intervention study
AppSalt: an application-based programme to reinforce and maintain lower salt intake
ASC: action on salt China
BP: blood pressure
CBPR: the community-based participatory research model
CCHE: the Chinese Centre for Health Education
CVD: cardiovascular disease
COVID-19: Corona Virus Disease 2019
cRCT: cluster randomized controlled trials
DELISH: a Digital Education to Limit Salt in the Home
EduSaltS: scaling-up research named school-based education programme to reduce salt Scaling-up in China
GACD: Global alliance for chronic diseases
GUIDED: Guidance for Reporting Intervention Development Studies in Health Research
HBM: health belief model and social learning theory
HPSI: health promoting school initiative
KAP: knowledge, attitude and practice
mHealth: mobile health
NCDs: non-communicable diseases
QUML: Queen Mary University of London
RCT: randomized controlled trial
School-EduSaltS: school-based education programme to reduce salt intake in children and their families
SMASH: The Shandong Ministry of Health Action on Salt Reduction and Hypertension
TGI: the George Institute for Global Health, China
WHO: World Health Organization

## References

[1] Shao S Hua Y Yang Y Liu X Fan J Zhang A. Salt reduction in China: a state-of-the-art review. Risk Manag Healthc Policy. (2017) 10:17–28. doi: 10.2147/rmhp.S75918, PMID: 28260957 PMC5328139

[2] He FJ MacGregor GA. A comprehensive review on salt and health and current experience of worldwide salt reduction Programmes. J Hum Hypertens. (2009) 23:363–84. doi: 10.1038/jhh.2008.144, PMID: 19110538

[3] World Health Organization. Salt reduction (2020). Available from: https://www.who.int/news-room/fact-sheets/detail/salt-reduction

[4] He FJ Zhang P Luo R Li Y Sun Y Chen F. App based education Programme to reduce salt intake (Appsalt) in schoolchildren and their families in China: parallel cluster randomised controlled trial. BMJ. (2022) 376:e066982. doi: 10.1136/bmj-2021-066982, PMID: 35140061 PMC8826455

[5] Zhou M Wang H Zeng X Yin P Zhu J Chen W. Mortality, morbidity, and risk factors in China and its provinces, 1990-2017: a systematic analysis for the global burden of disease study 2017. Lancet. (2019) 394:1145–58. doi: 10.1016/s0140-6736(19)30427-1, PMID: 31248666 PMC6891889

[6] Bi Z Liang X Xu A Wang L Shi X Zhao W. Hypertension prevalence, awareness, treatment, and control and sodium intake in Shandong Province, China: baseline Results from Shandong-Ministry of Health action on salt reduction and hypertension (smash), 2011. Prev Chronic Dis. (2014) 11:E88. doi: 10.5888/pcd11.130423, PMID: 24854239 PMC4032056

[7] Xu A Ma J Guo X Wang L Wu J Zhang J. Association of a Province-Wide Intervention with salt intake and hypertension in Shandong Province, China, 2011-2016. JAMA Intern Med. (2020) 180:877–86. doi: 10.1001/jamainternmed.2020.0904, PMID: 32338717 PMC7186913

[8] National Health Commission of the People’s Republic of China. An outline for the ‘National Healthy Lifestyle Programs‘initiative (2017). Available from: http://www.nhc.gov.cn/jkj/s5878/201704/e73c1934c7f84c709e445f01bf832b17.shtml.

[9] Ali SH Luo R Li Y Liu X Tang C Zhang P. Application of Mobile health technologies aimed at salt reduction: systematic review. JMIR Mhealth Uhealth. (2019) 7:e13250. doi: 10.2196/13250, PMID: 30994467 PMC6492062

[10] He FJ Zhang P Li Y MacGregor GA. Action on salt China. Lancet. (2018) 392:7–9. doi: 10.1016/s0140-6736(18)31138-3 30047401

[11] He FJ Wu Y Ma J Feng X Wang H Zhang J. A school-based education Programme to reduce salt intake in children and their families (school-Edusalt): protocol of a cluster randomised controlled trial. BMJ Open. (2013) 3:e003388. doi: 10.1136/bmjopen-2013-003388, PMID: 23864214 PMC3717470

[12] He FJ Zhang P Luo R Li Y Chen F Zhao Y. An application-based Programme to reinforce and maintain lower salt intake (Appsalt) in schoolchildren and their families in China. BMJ Open. (2019) 9:e027793. doi: 10.1136/bmjopen-2018-027793, PMID: 31272977 PMC6615780

[13] Tan M He FJ Wang C MacGregor GA. Twenty-four-hour urinary sodium and potassium excretion in China: a systematic review and meta-analysis. J Am Heart Assoc. (2019) 8:e012923. doi: 10.1161/JAHA.119.012923 31295409 PMC6662145

[14] Trieu K Neal B Hawkes C Dunford E Campbell N Rodriguez-Fernandez R. Salt reduction initiatives around the world–a systematic review of Progress towards the global target. PLoS One. (2015) 10:e0130247. doi: 10.1371/journal.pone.0130247, PMID: 26201031 PMC4511674

[15] Du S Wang H Zhang B Popkin BM. Dietary potassium intake remains low and sodium intake remains high, and Most sodium is derived from home food preparation for Chinese adults, 1991-2015 trends. J Nutr. (2020) 150:1230–9. doi: 10.1093/jn/nxz332, PMID: 31909790 PMC7198305

[16] Liu X Tian H. An intervention study on smoking in children and their family members in Chengdu. China Health Manag. (2004) 2:117–8.

[17] Fornari LS Giuliano I Azevedo F Pastana A Vieira C Caramelli B. Children first study: how an educational program in cardiovascular prevention at school can improve Parents’ cardiovascular risk. Eur J Prev Cardiol. (2013) 20:301–9. doi: 10.1177/2047487312437617, PMID: 22345689

[18] Gunawardena N Kurotani K Indrawansa S Nonaka D Mizoue T Samarasinghe D. School-based intervention to enable school children to act as change agents on weight, physical activity and diet of their mothers: a cluster randomized controlled trial. Int J Behav Nutr Phys Act. (2016) 13:45. doi: 10.1186/s12966-016-0369-7, PMID: 27048282 PMC4822262

[19] He FJ Wu Y Feng XX Ma J Ma Y Wang H. School based education Programme to reduce salt intake in children and their families (school-Edusalt): cluster randomised controlled trial. BMJ. (2015) 350:h770. doi: 10.1136/bmj.h770, PMID: 25788018 PMC4364292

[20] Li X Jan S Yan LL Hayes A Chu Y Wang H. Cost and cost-effectiveness of a school-based education program to reduce salt intake in children and their families in China. PLoS One. (2017) 12:e0183033. doi: 10.1371/journal.pone.0183033, PMID: 28902880 PMC5597122

[21] Chen J Tian Y Liao Y Yang S Li Z He C. Salt-restriction-spoon improved the salt intake among residents in China. PLoS One. (2013) 8:e78963. doi: 10.1371/journal.pone.0078963, PMID: 24244395 PMC3823994

[22] Payne Riches S Piernas C Aveyard P Sheppard JP Rayner M Albury C. A Mobile health salt reduction intervention for people with hypertension: results of a feasibility randomized controlled trial. JMIR Mhealth Uhealth. (2021) 9:e26233. doi: 10.2196/26233, PMID: 34673535 PMC8569539

[23] Zhang P He FJ Li Y Li C Wu J Ma J. Reducing salt intake in China with “action on salt China” (Asc): protocol for campaigns and randomized controlled trials. JMIR Res Protoc. (2020) 9:e15933. doi: 10.2196/15933, PMID: 32271155 PMC7180507

[24] Sun Y Li Y He FJ Liu H Sun J Luo R. Process evaluation of an application-based salt reduction intervention in school children and their families (Appsalt) in China: a mixed-methods study. Front Public Health. (2022) 10:744881. doi: 10.3389/fpubh.2022.744881, PMID: 35359790 PMC8963959

[25] Global alliance for chronic diseases. School-Based Education Programme to Reduce Salt: Scaling-up in China (Edusalts) (2022). Available at: https://www.gacd.org/community/research-network/projects/su 14.

[26] Zhang X Hu X Ma J Zhang P Li Y Luo R. Cluster randomised controlled trial of home cook intervention to reduce salt intake in China: a protocol study. BMJ Open. (2020) 10:e033842. doi: 10.1136/bmjopen-2019-033842, PMID: 32385058 PMC7228508

[27] Du W Zhang P Zhang J Li Y He FJ Zhang X. Sodium reduction in restaurant food: a randomized controlled trial in China. Nutrients. (2022) 14. doi: 10.3390/nu14245313, PMID: 36558472 PMC9781955

[28] Neal B Wu Y Feng X Zhang R Zhang Y Shi J. Effect of salt substitution on cardiovascular events and death. N Engl J Med. (2021) 385:1067–77. doi: 10.1056/NEJMoa2105675, PMID: 34459569

[29] The CPC Central Committee and the State Council. Outline of the “healthy China 2030” *Plan* (2016). Available at: http://www.gov.cn/zhengce/2016-10/25/content_5124174.htm. Accessed March 23, 2023.

[30] Aarons GA Hurlburt M Horwitz SM. Advancing a conceptual model of evidence-based practice implementation in public service sectors. Admin Pol Ment Health. (2011) 38:4–23. doi: 10.1007/s10488-010-0327-7, PMID: 21197565 PMC3025110

[31] World Health Organization. Nine steps for developing a scaling-up strategy (2010). Available at: https://apps.who.int/iris/handle/10665/44432. Accessed April 1, 2021.

[32] Cheikh Ismail L Hashim M Jarrar AH Mohamad MN Al Daour R Al Rajaby R. Impact of a nutrition education intervention on salt/sodium related knowledge, attitude, and practice of university students. Front Nutr. (2022) 9:830262. doi: 10.3389/fnut.2022.830262, PMID: 35284451 PMC8914224

[33] Aparna P Salve HR Anand K Ramakrishnan L Gupta SK Nongkynrih B. Knowledge and behaviors related to dietary salt and sources of dietary sodium in North India. J Family Med Prim Care. (2019) 8:846–52. doi: 10.4103/jfmpc.jfmpc_49_19, PMID: 31041212 PMC6482771

[34] Ghimire K Adhikari TB Rijal A Kallestrup P Henry ME Neupane D. Knowledge, attitudes, and practices related to salt consumption in Nepal: findings from the community-based Management of non-Communicable Diseases Project in Nepal (Cobin). J Clin Hypertens (Greenwich). (2019) 21:739–48. doi: 10.1111/jch.13544, PMID: 31026125 PMC8030483

[35] Chen J Liao Y Li Z Tian Y Yang S He C. Determinants of salt-restriction-spoon using behavior in China: application of the health belief model. PLoS One. (2013) 8:e83262. doi: 10.1371/journal.pone.0083262, PMID: 24376675 PMC3869780

[36] Janz NK Becker MH. The health belief model: a decade later. Health Educ Q. (1984) 11:1–47. doi: 10.1177/109019818401100101, PMID: 6392204

[37] Ma Y Feng X Ma J He FJ Wang H Zhang J. Social support, social network and salt-reduction Behaviours in children: a substudy of the school-Edusalt trial. BMJ Open. (2019) 9:e028126. doi: 10.1136/bmjopen-2018-028126, PMID: 31203245 PMC6589018

[38] Zhang L Zhao F Zhang P Gao J Liu C He FJ. A pilot study to validate a standardized one-week salt estimation method evaluating salt intake and its sources for family members in China. Nutrients. (2015) 7:751–63. doi: 10.3390/nu7020751, PMID: 25621504 PMC4344558

[39] Eyles H McLean R Neal B Jiang Y Doughty RN McLean R. A salt-reduction smartphone app supports lower-salt food purchases for people with cardiovascular disease: findings from the Saltswitch randomised controlled trial. Eur J Prev Cardiol. (2017) 24:1435–44. doi: 10.1177/2047487317715713, PMID: 28631933

[40] National Health and Family Planning Commission. Guide to the generation and dissemination of popular health science information (2015). Available at: http://www.nhc.gov.cn/xcs/s3581/201508/5fe32b5a1a8243e2bd819f9eeebfd8b1.shtml. Accessed December 1, 2022.

[41] Chinese Center for Health Education. Resource Bank for Health Promotion and Health Education. Available at: http://www.rbhp.org.cn:81/portal/index.htm. Accessed December 1, 2022.

[42] Duncan E O’Cathain A Rousseau N Croot L Sworn K Turner KM. Guidance for reporting intervention development studies in Health Research (Guided): an evidence-based consensus study. BMJ Open. (2020) 10:e033516. doi: 10.1136/bmjopen-2019-033516, PMID: 32273313 PMC7245409

[43] The George institute for Global Health. Edusalts management platform (2021). Available at: https://www.myrct.cn/edusalt_admin/web_manage/homepage_v=1.22.html. Accessed July 24, 2022.

[44] Ministry of Education. Guidelines for health education in primary and secondary schools (2008). Available from: http://www.gov.cn/gongbao/content/2009/content_1310690.htm. December 1, 2022.

[45] Hoddinott P. A new era for intervention development studies. Pilot Feasibility Stud. (2015) 1:36. doi: 10.1186/s40814-015-0032-0, PMID: 27965814 PMC5153779

[46] Grimes CA Booth A Khokhar D West M Margerison C Campbell KJ. Digital education to limit salt in the home (Delish) program improves knowledge, self-efficacy, and behaviors among children. J Nutr Educ Behav. (2018) 50:547–54. doi: 10.1016/j.jneb.2018.04.002, PMID: 29886898

[47] Grimes CA Booth A Khokhar D West M Margerison C Campbell K. The development of a web-based program to reduce dietary salt intake in schoolchildren: study protocol. JMIR Res Protoc. (2017) 6:e103. doi: 10.2196/resprot.7597, PMID: 28566266 PMC5471360

[48] Bouterakos M Booth A Khokhar D West M Margerison C Campbell KJ. A qualitative investigation of school age children, their parents and school staff on their participation in the digital education to limit salt in the home (Delish) program. Health Educ Res. (2020) 35:283–96. doi: 10.1093/her/cyaa015, PMID: 32632439

[49] Liu Z Gao P Gao AY Lin Y Feng XX Zhang F. Effectiveness of a multifaceted intervention for prevention of obesity in primary school children in China: a cluster randomized clinical trial. JAMA Pediatr. (2022) 176:e214375. doi: 10.1001/jamapediatrics.2021.4375, PMID: 34747972 PMC8576631

[50] Perin MS São-João T Gallani M Agbadje TT Rodrigues RCM Cornélio ME. A Mobile phone app intervention to promote healthy salt intake among adults: protocol for a randomized controlled study. JMIR Res Protoc. (2022) 11:e37853. doi: 10.2196/37853, PMID: 35767347 PMC9280466

[51] Jarrar AH Al Dhaheri AS Lightowler H Cheikh Ismail L Al-Meqbaali F Bataineh MF. Using digital platform approach to reduce salt intake in a sample of Uae population: an intervention study. Front Public Health. (2022) 10:860835. doi: 10.3389/fpubh.2022.860835, PMID: 35685760 PMC9172248

[52] Kerry SM Emmett L Micah FB Martin-Peprah R Antwi S Phillips RO. Rural and semi-urban differences in salt intake, and its dietary sources, in Ashanti. West Africa Ethn Dis. (2005) 15:33–9. 15720047

[53] Ghimire K Mishra SR Satheesh G Neupane D Sharma A Panda R. Salt intake and salt-reduction strategies in South Asia: from evidence to action. J Clin Hypertens (Greenwich). (2021) 23:1815–29. doi: 10.1111/jch.14365, PMID: 34498797 PMC8678780

[54] Batcagan-Abueg AP Lee JJ Chan P Rebello SA Amarra MS. Salt intakes and salt reduction initiatives in Southeast Asia: a review. Asia Pac J Clin Nutr. (2013) 22:490–504. doi: 10.6133/apjcn.2013.22.4.04, PMID: 24231008

[55] Brown IJ Tzoulaki I Candeias V Elliott P. Salt intakes around the world: implications for public health. Int J Epidemiol. (2009) 38:791–813. doi: 10.1093/ije/dyp139, PMID: 19351697

[56] Anderson CA Appel LJ Okuda N Brown IJ Chan Q Zhao L. Dietary sources of sodium in China, Japan, the United Kingdom, and the United States, women and men aged 40 to 59 years: the Intermap study. J Am Diet Assoc. (2010) 110:736–45. doi: 10.1016/j.jada.2010.02.007, PMID: 20430135 PMC4308093

[57] Davé A Rentel M King M Babb R Nausedaite R. Case study prepared by Technopolis group. Available at: https://www.gacd.org/perch/resources/china-salt-reduction-case-study.pdf. Accessed January 2, 2023.

