# Supplementary material for: An mHealth-based school health education system designed to scale up salt reduction in China (EduSaltS): A development and preliminary implementation study
Source: Front Nutr. 2023 Apr 17;10:1161282. doi: 10.3389/fnut.2023.1161282 (PMC10149706; doi:10.3389/fnut.2023.1161282)
Supplement: Supplementary file 1 [file Data_Sheet_1.pdf]

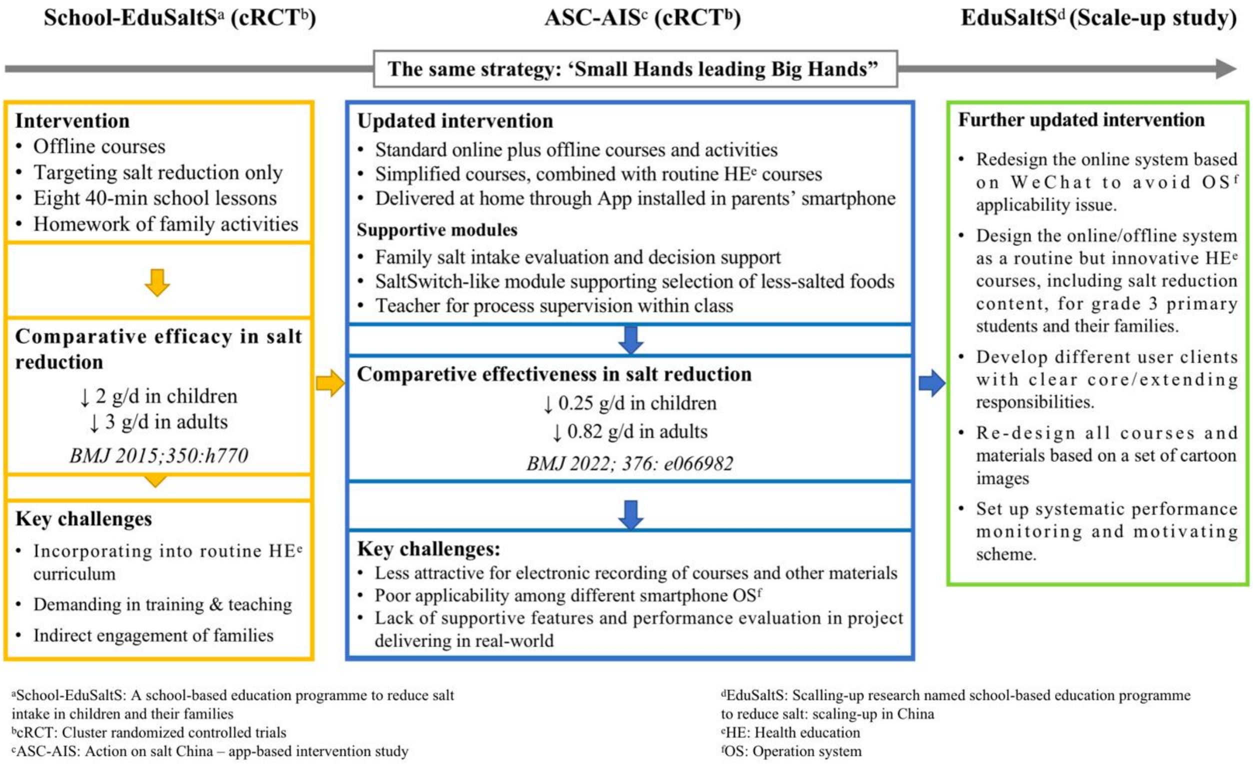

Figure 1. The evolution of interventions from School-EduSalt, AppSalt to EduSaltS

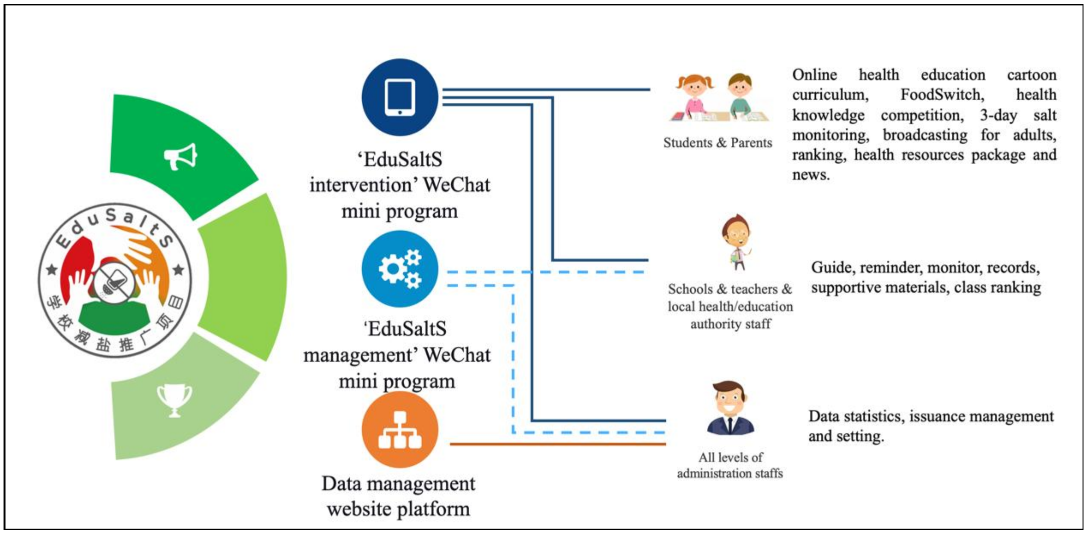

Figure 2. Three electronic components of the 'EduSaltS' system

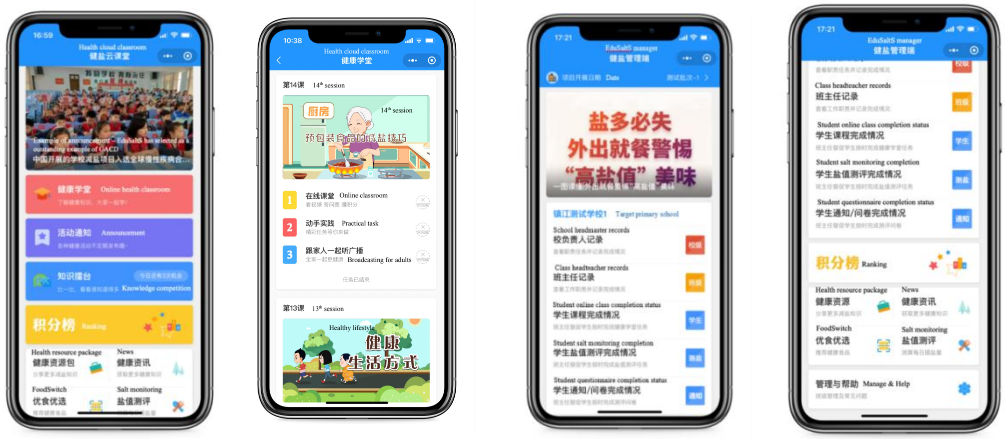

Home screen | Health education course | Home screen

Health Cloud Classroom
- The children/parents' client

EduSaltS Manager
- The teachers/supervisors' client

Figure 3. The main features of 'Health Cloud Classroom' and 'EduSaltS Manager'

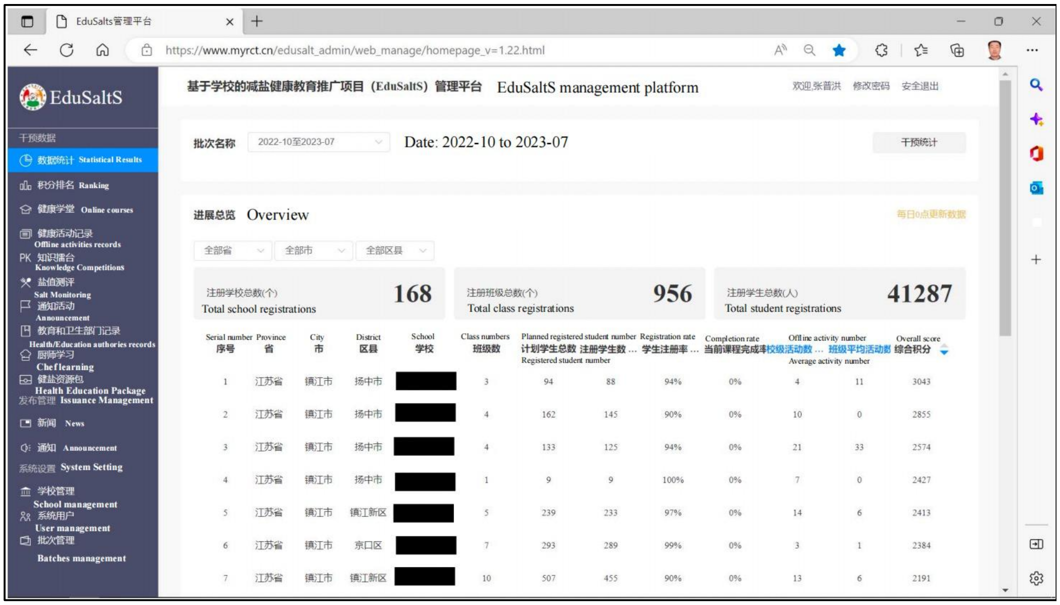

Figure 4. The web based EduSaltS management platform for statistical reports and system setting
